# Nursing Interventions and Care Strategies for Patients with Coronary Heart Disease: A Comprehensive Review

**DOI:** 10.31661/gmj.v12i.2994

**Published:** 2023-08-19

**Authors:** Yangyan Shan, Jun Chen, Siwen Zhou, Guangxue Wen

**Affiliations:** ^1^ Department of Hemodialysis Room, Funan County Hospital of Traditional Chinese Medicine, Funan, Anhui 236300, China; ^2^ Department of Nephrology, Funan County Hospital of Traditional Chinese Medicine, Funan, Anhui 236300, China

**Keywords:** Coronary Heart Disease, Nursing Care, Patient Education, Quality of Life, Cardiac Rehabilitation

## Abstract

Cardiovascular diseases are a major cause of death worldwide, and coronary heart disease (CHD) is a prevalent cardiovascular condition and a significant health burden for the population. In this disease, insufficient blood flow to the heart due to plaque accumulation in the coronary arteries causes chest pain, heart attack, and even death. So, it is vital to identify risk factors, prevention, appropriate treatment, and rehabilitation. Nurses play an indispensable role in managing and caring for patients with CHD. Indeed, they possess a deep understanding of the disease and its complexities, enabling them to provide comprehensive care to patients. Nurses monitor vital signs, administer medications, and perform diagnostic tests, ensuring patients receive timely and appropriate interventions. They also educate patients and their families about CHD, emphasizing lifestyle modifications, medication adherence, and self-care practices. Moreover, nurses offer emotional support, guiding patients through the physical and psychological challenges associated with CHD. Their expertise, compassion, and dedication significantly improve patient outcomes and overall quality of life. Nurses are responsible for assessing, diagnosing, and counseling patients on how to manage their disease, making them the front line of defense in preventing and addressing this serious condition. In the current study, we reviewed the literature to consider the best practices and emerging trends in nursing interventions and care strategies for patients with CHD.

## Introduction

Coronary heart disease (CHD) is a prevalent and life-threatening condition that requires diligent nursing care [1][2]. Nursing care plays a crucial role in the early detection and prevention of CHD. Also, they conduct thorough assessments, monitor vital signs, and evaluate risk factors to identify individuals at risk for developing the disease [3].

Through health promotion and education, nurses empower patients with knowledge about lifestyle modifications, such as adopting a heart-healthy diet, engaging in regular exercise, and managing stress. By implementing preventive measures, nurses can potentially reduce the incidence of CHD and its associated complications [3][4][5]. Also, they collaborate with healthcare teams to develop individualized care plans for medication management, cardiac rehabilitation (CR), and regular follow-up visits [6][7][8][9]. Moreover, they monitor patients’ symptoms, administer medications, and educate them about their prescribed medications’ purpose, dosage, and potential side effects [6].

Nurses provide ongoing support and counseling, addressing patients’ concerns, promoting adherence to treatment plans, and encouraging healthy lifestyle choices. Also, they closely monitor patients’ progress and can identify any changes or complications, facilitating timely interventions to prevent further deterioration [10][11][12][13][14].

Moreover, nursing care plays a vital role in the emotional and psychological support of patients. The diagnosis of CHD could trigger feelings of fear, anxiety, and stress, which hurt the mental well-being of patients [15][16]. Hence, nurses provide a compassionate and empathetic presence, actively listen to patients’ concerns, and address their emotional needs. In addition, they offer guidance and coping strategies, promoting resilience and a positive mindset [4]. Moreover, nurses facilitate support groups or refer patients to appropriate resources, fostering a sense of community and helping patients navigate the emotional challenges associated with the disease [7]. By providing holistic care, nurses contribute significantly to enhancing patients’ overall well-being and improving their quality of life (QoL) in the face of disease [17][18].

Regarding the important significant impact of nursing care on patients with CHD, we reviewed the current literature on the role of nurses in the management and emerging trends in nursing interventions for CHD.

1. Pathophysiology and Clinical Presentation of CHD

The development of CHD is primarily attributed to atherosclerosis, inflammation, and oxidative stress [19]. Atherosclerosis is a chronic inflammatory disease related to the lipid profile and caused by plaque formation in the coronary arteries. These plaques obstruct blood flow causing the onset of CHD. Low-density lipoprotein (LDL) cholesterol oxidation significantly contributes to atherosclerosis induction and progression [19][20][21].

So a high intake of saturated fats, trans fats, and cholesterol can contribute to atherosclerosis [22][23]. Furthermore, psychiatric disorders such as depression, anxiety, and stress are notable risk factors for CHD. So, several risk factors are identified for CHD, including an unhealthy diet, physical inactivity, smoking, stress, and genetic predisposition [22][23].

CHD can present with various symptoms and clinical manifestations, such as chest pain, shortness of breath, and fatigue [19]. However, in vital conditions, CHD presents with no symptoms at all, known as silent ischemia, and could result in more severe events [24]. Therefore, early detection and management of risk factors for CHD are critical to preventing its onset, progression, and treatment [25].

2. Risk Assessment and Patient Education

Comprehensive risk assessment tools are essential for identifying at-risk CHD patients and developing appropriate treatment plans [26]. Indeed, it empowers patients with knowledge of their risk factors and encourages them to actively make positive lifestyle changes and adhere to recommended treatments [26][27].

Effective patient education strategies include teaching resourcefulness, providing patient-centered education, using digital health strategies, assessing genetic counseling, and providing regular and ongoing communication and support [4][5].

Indeed, teaching resourcefulness is an effective strategy to improve mental disorders such as depression and stress [12][15][28]. Also, patient-centered education should be evidence-based and tailored to patients’ needs and preferences [29][30]. Consequently, digital health strategies facilitate education and support for patients with cardiovascular diseases (CVDs), including text messaging platforms, cellphone applications, and wearable devices [14][31][32][33]. Also, genetic counselors are responsible for caring for patients with CHD and exploring recalled education experiences can provide valuable insights [34]. In addition, regular and ongoing communication and support are crucial for ensuring that patients understand their condition, develop appropriate lifestyle modifications, and improve their QoL [7][25].

3. Nursing Assessment and Diagnosis

Assessment and diagnosis are essential to providing best practices for patients with CHD. Indeed, an appropriate nursing assessment includes a review and evaluation of medical records, a physical examination, mental health status, laboratory results, lifestyle, and dietary habits [8][35][36]. Hence, it could help nurses identify potential health risks and/or conditions and provide the basis for an individualized nursing care plan. Also, it helps to ensure that nursing interventions are tailored to meet the individual’s needs [8].

For instance, it has been shown that a personalized educational plan focused on health promotion could improve the QoL of patients with CHD [37]. In fact, this plan aims to modify or enhance a cardiac-healthy lifestyle and facilitate strategies to promote patient empowerment [37][38]. Also, nurses can provide patients with a food frequency questionnaire to assess their dietary habits and identify areas for improvement [37]. Mindfulness-based interventions have also reduced depression and stress in patients [37].

In other words, identifying and classifying nursing diagnoses related to CHD are vital for providing effective and targeted patient care [36][39].

One important CHD-related nursing diagnosis is decreased cardiac output [8][23]. This diagnosis is made when the heart’s pumping ability is compromised, leading to inadequate blood supply [35]. Appropriate interventions to improve cardiac output include administering medications to enhance heart function [36]. In addition, they promote physical activity within safe limits and educate patients on lifestyle modifications to reduce cardiac workload [41].

Another significant nursing diagnosis is the risk for impaired tissue perfusion, which applies to patients at risk of inadequate blood flow to specific body tissues due to narrowed or obstruction of blood vessels [39]. In this case, nurses evaluate the patient’s peripheral pulses, skin color, temperature, and capillary refill time to determine tissue perfusion status [8][36].

Therefore, preventive measures such as monitoring and managing blood pressure and cholesterol levels, promoting smoking cessation, providing patient education on cardiac-healthy diet, and ensuring adherence to prescribed medications can reduce the risk of further tissue damage and improve overall perfusion in patients with CHD [35].

4. Pharmacological Management and Nursing Implications

The management of CHD requires a multifaceted approach to treatment. Medications are critical in managing symptoms, preventing complications, and improving overall cardiac health [43][44][45]. The commonly prescribed CHD medications are presented in Table-1. Variations in drugs prescribed for CHD are based on individual patient characteristics, such as symptoms, risk factors, and overall cardiac disorders [45]. Medication dosages and combinations are tailored to each patient’s needs, and regular monitoring as well as follow-up with healthcare providers, are crucial for optimal CHD management [8][45].

Nurses are responsible for ensuring the safe administration and monitoring of medications for CHD and should consider the following aspects:

4.1. Patient Education

Nurses are responsible for educating patients on the importance of taking medications as prescribed, including the correct dosage, frequency, and timing [4]. Also, patients should be informed about potential side effects and adverse reactions. Hence, this education should be clear, concise, and appropriate for the patient’s level of understanding [46].

4.2. Medication Reconciliation

Nurses ensure patients’ medication lists are accurate and up-to-date, including prescription medications, over-the-counter medications, and herbal supplements [45]. This process involves obtaining a comprehensive medication history and reconciling any discrepancies [45].

4.3. Medication Administration

Nurses administer medications as prescribed based on the "six rights" of medication administration (i.e., right patient, right medication, right dose, right route, right time, and right documentation). This process should be performed with attention to detail and adherence to established protocols [47].

4.4. Monitoring the Adverse Effects

Nurses monitor patients for potential side effects and adverse reactions to medications. This includes changes in vital signs, laboratory values, and symptoms (e.g., dizziness, nausea, and rash) [8]. Hence, nurses should promptly report any adverse effects to the healthcare team [40].

4.5. Compliance Monitoring

The patients’ compliance with medication regimens, including assessing for barriers to adherence and providing support and education to improve compliance, is another nurses’ responsibility [46].

This process should be done in collaboration with the patient and the healthcare team [48].

4.6. Collaboration with the Healthcare Team

Nurses collaborate with other healthcare professionals, including physicians, pharmacists, etc. [9]. This collaboration is essential to ensure patients receive appropriate and effective medication management [49].

4.7. Extended Nursing

Extended nursing interventions, such as continuous and comprehensive interventions, have been shown to improve medication compliance and promote patient recovery through appropriate behavior [39][46]. These interventions involve ongoing support and education for patients and collaboration with the healthcare team [48].

**Table:** **Table1.** Common Prescribed Medication for CHD

**Medications**	**Functions**	**Recommended for**
**Beta-blocker**	Reduce workload on the heart by declining the heart rate and reducing blood pressure	Decrees the risk of heart attack and complications
**ACE inhibitors and ARB**	Lower blood pressure and reduce risk of heart attack and complications	Patients who have high blood pressure or heart failure
**Antiplatelet**	Prevent blood clot formation	Reduces the risk of heart attack and stroke
**Statins**	Lower serum cholesterol levels and reduce the risk of heart attack and stroke	Reduces cholesterol levels
**Anticoagulants**	Prevent blood clots formation in veins and arteries	Patients who have a high risk of blood clots
**Nitroglycerin**	Relieve chest pain vasodilation and increase blood flow to the heart	Reduces experiencing angina
**Calcium channel blocker**	Vasodilation and reduced blood pressure	Patients who have high blood pressure or angina

**ACE:**Angiotensin-converting enzyme;**ARB:**Angiotensin II receptor blockers

5. CR and Lifestyle Modifications

These interventions are essential for improving CVDs fitness, reducing risk factors, enhancing psychological well-being, and promoting long-term health in patients with CHD [32][50]. The CVDs rehabilitation phase I, which involves education and counseling, exercise and physical activity, breathing exercises, chest physiotherapy, breathing muscle stretching exercises, and gradual mobilization, is essential to the treatment of patients with CHD [51][52]. Nurses are involved in various aspects of CR, including patient assessment, education, and psychosocial support. Also, they ensure continuity of care across different healthcare settings and provide informational, management, and relational continuity to ensure coherent, logical, and timely services [53][54][55].

Nurses play a vital role in patient education, helping patients understand their condition, treatment options, and lifestyle modifications necessary for optimal recovery [3][53]. They also assess and monitor patients’ progress throughout the CR program, including tracking attendance at CR sessions, evaluating health-related self-efficacy, and identifying any barriers to program adherence [7][53].

Also, they could collaborate with other healthcare professionals to develop and implement individualized CR plans for each patient [48]. Indeed, they can improve CR program enrollment and adherence rates by implementing evidence-based strategies at the institutional level, facilitating patient referrals to CR programs, providing ongoing support and encouragement, and addressing any barriers to participation [53]. In addition, they could help design and implement hybrid CR programs, which combine facility-based CR with virtual and/or remote CR, to increase accessibility and convenience for patients [56].

6. Psychosocial Support and Patient Counseling

Patients with CHD must maintain their mental well-being because it directly affects their disease progression and overall QoL [15].

Several studies demonstrated that psychological interventions, such as patient education, positive psychology-based approaches, and cognitive-behavioral therapy (CBT) improve mental health and well-being in CHD patients [27][46][57][58][59][60].

Also, support from family plays an essential role in facilitating these patients’ psychological adjustments and improving their QoL [10][15]. Patients with CHD may also be able to improve health-promoting behaviors through psychological interventions, counseling (e.g., spiritual and genetic), and improving self-control [7].

de Eston et al. [61] demonstrated no relationship between religious, spiritual, and existential well-being and CHD.

Also, Li et al. [62] found that self-control indirectly and positively predicted the level of health-promoting behavior in patients with CHD, suggesting that interventions aimed at improving self-control may be beneficial for these patients.

7. Nursing Care During Acute Coronary Syndrome (ACS)

Nursing interventions and management strategies are crucial for patient care and recovery during acute coronary events, specifically ST-segment elevation myocardial infarction (STEMI) and non-ST-segment elevation acute coronary syndrome (NSTE-ACS) [63][64].

Initial assessment and monitoring are essential for identifying risk factors, evaluating chest pain, and interpreting electrocardiogram results [65]. Continuous monitoring of vital signs, including blood pressure, heart rate, and oxygen saturation are crucial. Hence, administering appropriate medications is one of the important interventions for managing acute coronary events [8][66].

Patients with STEMI need rapid reperfusion therapy to minimize myocardial damage and improve outcomes. Also, different coronary revascularization strategies may be employed depending on the patient’s condition and hospital protocols [45]. These strategies include early and/or delayed invasive or conservative strategies [45].

Nursing interventions and management strategies during acute coronary events, such as STEMI and NSTE-ACS, involve initial assessment, monitoring, medication administration, reperfusion therapy, education and counseling, management of complications, and coordination of care [66]. These interventions are essential for providing optimal care and improving patient outcomes [8][66].

Moreover, emergency response protocols and immediate care considerations for nursing care in patients with CHD are essential to ensure proper management and reduce complications [67].

Therefore, nurses should be aware of the typical symptoms of CHD and promptly assess any sudden changes in the patient’s condition, activate the emergency response team, initiate basic life support, administer emergency medications, continuously monitor and evaluate the patient, provide emotional support, and collaborate with the healthcare team [39][47][67].

8. Advances in Nursing Technology and Digital Health

Innovative technologies have the potential to significantly impact nursing care for patients with CHD [31][33][35]. These technologies improve patient outcomes, enhance self-management, and facilitate better communication between patients and healthcare providers [55][68]. Some of these technologies are as follows:

8.1. Electronic Health Records (EHRs)

EHRs allow nurses to access and document patient information electronically, promoting seamless communication and continuity of care [31]. Nurses can efficiently retrieve and update patient data and collaborate with other healthcare providers, ensuring comprehensive and coordinated care for patients with CHD [31].

8.2. Mobile Health (mHealth) Applications

The mHealth applications designed for CHD management able nurses to deliver personalized care and support to patients remotely [68]. These applications provide education on medications, lifestyle modifications, and symptom management [69].

Also, nurses are able to remotely monitor patients’ vital signs, track medication adherence, and provide timely feedback and interventions. Hence, mHealth applications could facilitate patient engagement and self-management as well as improve overall outcomes [70][71].

8.3. Remote Patient Monitoring

By introducing remote monitoring technologies, such as wearable devices and sensors, nurses enable to track patients’ vital signs, activity levels, and symptoms remotely [9]. Real-time data transmission allows nurses to identify early warning signs of cardiac events, such as changes in heart rate and blood pressure [12].

Also, nurses could proactively intervene, guide, and escalate care when necessary, which leads to preventing complications and reducing hospital readmissions [9][12][14].

8.4. Telehealth and Virtual Visits

With telehealth platforms, nurses can conduct virtual patient visits, improving care access and reducing travel barriers [12]. Also, they remotely assess symptoms, conduct medication reviews, and provide counseling on lifestyle modifications [9][12]. Also, telehealth facilitates regular patient follow-ups, enhances patient-provider communication, and promotes continuity of care and patient satisfaction [9][12][14].

8.5. Clinical Decision Support Systems (CDSSs)

CDSSs are software that provides evidence-based recommendations and alerts to guide nurses in making informed clinical decisions [72]. In CHD care, CDSSs assist with medication dosing calculations, identifies potential drug interactions, and offer treatment guidelines based on patient-specific factors [73]. Therefore, these systems could promote standardized, safe, and efficient nursing practices, reducing error risk and improving patient outcomes [72][73].

8.6. Health Education, Augmented Reality (AR), and Virtual Reality (VR)

Nurses can use VR to create immersive and engaging educational experiences for patients with CHD [74][75].

AR and VR simulations can demonstrate the effects of lifestyle choices, teach medication administration techniques, and promote adherence to treatment plans. By using VR, nurses can enhance health education and empower patients to take active self-care roles [74][75][76][77].

8.7. Artificial intelligence (AI)

AI encompasses various methodologies to impart human-like cognitive abilities, such as reasoning, communication, learning, and decision-making to computers [78][79]. Numerous subfields of AIs include robotics, machine learning, deep learning, and natural language processing [78][79][80]. AI and nursing care integration in CHD management has shown promising results in various aspects, such as decision-making, monitoring, and diagnosis [79]. AI deep learning techniques have also been applied to predict possible complications and improve clinical nursing quality for patients with CHD [81].

9. Interdisciplinary Collaboration and Care Coordination

A growing body of literature highlights the positive impact of interdisciplinary teamwork on patient outcomes and quality of care for patients with CHD [49][82]. Effective collaboration among healthcare professionals leads to better adherence to treatment guidelines, improved medication management, and enhanced patient education [83].

Williams et al. [49] showed that interdisciplinary teamwork in CHD management reduces hospital readmissions, improves patient satisfaction, and increases patient empowerment. These findings underscore the significant role of interdisciplinary collaboration in ensuring comprehensive and patient-centered care [49]. Therefore, fostering collaborative relationships among healthcare professionals from different disciplines is essential for optimizing CHD management and enhancing patient outcomes [49].

Effective communication and collaboration require strategies among healthcare professionals to ensure the highest possible outcomes for patients with CHD [83].

One strategy is to use EHRs to facilitate communication and collaboration that provide a central location for healthcare professionals to access patient information [84]. It is helpful to ensure all healthcare professionals involved in the patient’s care have access to the same information, reducing errors as well as improving patient outcomes [84][85]. Another strategy is establishing regular team meetings allowing healthcare professionals to discuss patient care, share information, and coordinate their efforts [85]. Regular meetings can also help to identify potential issues and challenges in patient care and facilitate problem-solving [86].

10. Ethical Considerations in CHD Nursing Care

Regarding CHD, healthcare professionals may face ethical challenges and dilemmas that require careful consideration. Informed consent is a critical ethical challenge for healthcare professionals when treating patients [87][88]. Invasive procedures such as angioplasty carry risks and potential complications. Hence, patients must fully understand the risks and benefits of these procedures before consenting [88][89].

Cultural and/or religious beliefs may also impact patients’ decision-making processes, and healthcare professionals must provide culturally sensitive care that respects these beliefs [65][89].

Another ethical challenge in CHD care is resource allocation, a chronic condition requiring ongoing management and treatment, but healthcare resources are limited [90]. Indeed, healthcare professionals must ensure that resources are used efficiently to provide the best care for patients. This may involve making difficult decisions about which patients should receive certain treatments or interventions [91].

It may also involve balancing the needs of individual patients with the broader healthcare system’s needs to ensure resources are used fairly and equitably [90][91]. So, healthcare professionals must have a strong understanding of ethical principles and values. Continuing education and training can help healthcare professionals develop these skills and stay up-to-date on the latest ethical issues and debates in CHD care [92]. Additionally, healthcare organizations can develop policies and procedures that guide ethical decision-making in CHD care [88][92].

Studies indicated that individuals with CHD frequently possess an inadequate understanding of their condition and available treatment choices [87].

This lack of knowledge can stem from multiple factors, such as low health literacy, limited healthcare access, and communication challenges between patients and healthcare providers [87][88]. To ensure that patients’ autonomy is respected and their wishes are honored, healthcare professionals must adopt a patient-centered approach to CHD care, which includes providing patients with comprehensive information about their condition, discussing the risks and benefits of different treatment options, and engaging in end-of-life discussions about advance care planning, resuscitation preferences, and palliative care options, while considering the impact of cultural or religious beliefs, family dynamics, and other social factors [93][94][95].

11. Future Directions and Implications for Nursing Practice

Emerging trends and research areas in CHD nursing care focus on optimizing care delivery and improving patient outcomes as follows:

11.1. Integrated Nursing Care Based on the Medical Alliance Model

It is based on the medical alliance model that improves self-efficacy and -management abilities and reduces postoperative complications in patients with CHD after percutaneous coronary intervention (PCI) [6]. This approach involves collaboration between healthcare professionals, patients, and families to develop a comprehensive care plan that addresses the patient’s unique needs.

11.2. Omaha System-Based Continuing Care

It improves medication compliance and QoL and reduces major adverse cardiac events among patients with CHD after PCI [96]. This approach involves using a standardized assessment tool to identify patient needs and develop a care plan that addresses those needs [6][96][97][98].

11.3. Comprehensive Nursing Intervention Based on Self-Disclosure

It improves QoL and physical activity and reduces alexithymia, anxiety, depression, recurrence, and mortality among patients with CHD [99].

This approach involves encouraging patients to express their emotions and providing support to address their unique needs [99].

11.4. Comfort Care Based on the Collaborative Care Model

Patients with CHD benefit from this program by collaborating with healthcare professionals, patients, and their families to create a care plan that addresses their physical, emotional, and spiritual needs [48].

11.5. Mind-Body-Spiritual Nursing Care Model

Although not specifically mentioned in the literature, the mind-body-spiritual nursing care model may enhance the emotional and spiritual abilities of patients with CHD [100].

11.6. Emerging Technologies Nursing Practice

Emerging technologies promise to transform nursing practice by enhancing efficiency, improving patient outcomes, and expanding the scope of care [14][33]. Innovations such as telehealth, wearable devices, AIs, and VR are revolutionizing the methods that nurses deliver healthcare services [14][33].

Telehealth enables remote consultations, monitoring, and care coordination, breaking down geographical barriers and increasing access to quality care [14]. Also, wearable devices provide real-time health data, empowering nurses to track and manage patient conditions more effectively [33]. Moreover, AIs applications support clinical decision-making, enabling personalized care plans and early detection of potential risks [78][80].

As mentioned before, VR offers immersive training experiences, allowing nurses to practice complex procedures in a safe and controlled environment [75]. As these technologies continue to advance, nurses must embrace them as valuable tools in their practice, fostering a new era of patient-centered care and improved healthcare outcomes [101][102].

## Conclusion

Practical nursing care is crucial for improving patient outcomes and enhancing the QoL for patients with CHD. Nursing assessment and diagnosis play a vital role in providing comprehensive patient care by systematically gathering and analyzing data to identify health-related problems and formulate appropriate interventions. Also, they ensure safe and effective medication use, promote and support CR programs, and facilitate lifestyle modifications.

In psychosocial support and patient counseling, nursing care involves establishing a therapeutic nurse-patient relationship, CBT, and enhancing overall well-being.

During ACS, nursing care encompasses timely assessment, vigilant monitoring, prompt intervention, and effective collaboration among interdisciplinary teams to provide comprehensive cardiac care, optimize patient outcomes, and prevent complications in this critical CVDs emergency.

Further research and practice development in CHD nursing care should focus on optimizing sedation protocols during PCI, developing tailored lifestyle interventions and patient education, and exploring the application of patient activation measures in CHD nursing care.

Moreover, future research should evaluate and challenge the use of telehealth, wearable devices, AI, and VR in nursing practice.

## Conflict of Interest

The authors declare that there are no conflicts of interest.
